# PF-04691502, a PI3K/mTOR Dual Inhibitor, Ameliorates AD-like Pathology in a Mouse Model of AD

**DOI:** 10.3390/cells14181474

**Published:** 2025-09-21

**Authors:** Marika Lanza, Rossella Basilotta, Antonella Caccamo, Giovanna Casili, Alberto Repici, Salvatore Oddo, Emanuela Esposito

**Affiliations:** Department of Chemical, Biological, Pharmaceutical and Environmental Sciences, University of Messina, Viale Ferdinando Stagno D’Alcontres 31, 98166 Messina, Italy; mlanza@unime.it (M.L.); rossella.basilotta@unime.it (R.B.); antonella.caccamo@unime.it (A.C.); alberto.repici@unime.it (A.R.); salvatore.oddo@unime.it (S.O.); emanuela.esposito@unime.it (E.E.)

**Keywords:** Alzheimer’s disease, PI3K/mTOR inhibitor, learning and memory, autophagy

## Abstract

Alzheimer’s disease (AD) is a neurodegenerative disorder that significantly impacts the lives of patients and their families. The pathological features of AD include the accumulation of amyloid-β (Aβ) and Tau, which disrupt neuronal function and communication, ultimately leading to neuronal loss and brain atrophy. Efforts to understand the molecular mechanisms underlying these pathological changes have led to advancements in diagnostic techniques and potential therapeutic interventions. However, the complexity of AD necessitates further research to develop more effective treatments and, ideally, preventive measures. Extensive research suggests that diminishing mTOR signaling increases lifespan and health span across various species. Increased PI3K/mTOR signaling has been linked to the progression of AD pathology, leading to neuronal degeneration and impairments in cognitive function. In this study, we explored the therapeutic potential of PF-04691502, a dual PI3K/mTOR inhibitor, in Alzheimer’s disease (AD)-like pathology using male and female B6.Cg-Tg(APPswe, PSEN1dE9)85Dbo/Mmjax mice (APP/PS1), a well-established transgenic model of AD. Eighteen-month-old APP/PS1 and wild-type mice received oral administration of PF-04691502 at a dose of 1 mg/kg for 12 weeks. Following the treatment period, spatial learning and memory were evaluated using the Morris water maze. Subsequently, the mice brains were collected for neuropathological and biochemical assessments. Our findings showed that PF-04691502 enhanced cognitive performance in APP/PS1 mice and significantly reduced insoluble Aβ accumulation in the brain. Mechanistically, these effects were associated with enhanced autophagy induction. Treatment with PF-04691502 increased the LC3-II/LC3-I ratio, upregulated Beclin-1, and elevated LAMP-2 levels, indicative of stimulated autophagosome formation and lysosomal activity. Overall, these preclinical results suggest that PF-04691502 holds promise as a potential therapeutic agent for AD and other aging-related neurodegenerative diseases involving mTOR pathway dysregulation.

## 1. Introduction

Alzheimer’s disease (AD) is one of the most common neurodegenerative diseases worldwide, affecting people’s lives by leading to a gradual loss of cognitive functions, memory, and daily abilities [1]. Globally, the WHO estimated that in 2023 about 55 million people aged 60 or older were affected by dementia, of which 40 million were thought to have AD [2,3]. AD is characterized by the accumulation of abnormally folded amyloid-β (Aβ) and Tau [4,5]. The former leads to the formation of extracellular plaques, while the latter leads to neurofibrillary tangles. The accumulation of plaques and tangles is associated with neuronal loss, brain atrophy and chronic inflammation [6,7].

Several risk factors have been associated with sporadic AD, including non-modifiable and modifiable ones. The former include gender, age, genetics and family history; the latter include physical activity, smoking, education, staying socially and mentally active, blood pressure and diet [8]. In contrast, a small number of AD cases are linked to mutations in one of three genes: the amyloid precursor protein (*APP*), presenilin 1 and 2 (*PS1* and *PS2*) [9,10].

The *APP* gene is located on chromosome 21 and encodes a transmembrane glycoprotein macromolecule of 770 amino acids [11]. APP is processed by three enzymes, α-, β-, and γ-secretase [12,13], producing fragments of different amino acid lengths. In particular, β- and γ-secretases initiate the amyloidogenic pathway leading to the formation of Aβ [14,15]. The *PS1* gene is located on chromosome 14 [16], and its homolog, *PS2*, is on chromosome 1 [17]. PS1 and PS2 constitute the catalytic subunit of the γ-secretase complex with several interacting substrates, including Notch, adhesion proteins and β-catenin. Although the pathogenetic mechanisms of these mutations are unclear, they seem to negatively affect the correct processing of APP, causing an increase in Aβ42 levels, thereby accelerating its accumulation [18,19].

Under physiological conditions, Tau binds to microtubules stabilizing their shape; in AD, this protein undergoes hyperphosphorylation, structural misfolding, and aggregation, resulting in neurofibrillary tangles [7,20]. It is becoming increasingly clear that multiplicities and complex cellular mechanisms are involved in the development of AD. In particular, one of the most important and challenging signaling pathways in this kind of dementia involves the phosphatidylinositol 3-kinase (PI3K), protein kinase B (Akt) and mammalian target of rapamycin (mTOR), components of a complex cell signaling pathway that regulates a wide range of biological processes, including those related to growth, survival, metabolism and response to growth factors and nutrients [21].

mTOR is one of the major modulators of autophagy and its activity is regulated by several signaling pathways, including those that detect the cell’s energy level and initiate or stop the production of proteins [22]. Autophagy is closely related to AD progression and it had been hypothesized that it could counteract and degrade the accumulation of Aβ and tau protein [23]. Several synthetic molecules have been designed to block multiple key points in this signaling pathway. Through the combination of structure-based drug design and optimization focused on physicochemical properties during the lead optimization phase, the compound 2-amino-8-[trans-4-(2-hydroxyethoxy)cyclohexyl]-6-(6-methoxypyridin-3-yl)-4-methylpyrido[2,3-d]pyrimidin-7(8H)-one (PF-04691502) was identified. This molecule demonstrated strong in vitro inhibitory activity against both PI3K and mTOR, showed high selectivity across kinases, and exhibited significant in vivo efficacy in a mouse xenograft tumor model [24]. PF-04691502 is presently being evaluated in clinical trials for its potential use in cancer therapy [25].

Given the involvement of the PI3K/mTOR pathway in AD pathogenesis, we sought to evaluate the effects of a 12-week daily oral treatment with PF-04691502 on AD-like pathology of 18-month-old APP/PS1 mice.

## 2. Materials and Methods

### 2.1. Materials

Unless otherwise indicated, all compounds were obtained from Sigma-Aldrich Company Ltd. (St. Louis, MO, USA). All other chemicals were of the highest commercial grade available. All stock solutions were prepared in non-pyrogenic saline (0.9% NaCl; Baxter, Rome, Italy).

### 2.2. Animals

Adult male and female B6.Cg-Tg(APPswe, PSEN1dE9)85Dbo/Mmjax mice (30–35 g; 18 months old at the beginning of the study), hereafter referred to as APP/PS1 mice, along with age- and weight-matched wild-type (WT) controls, with no signs of illness or abnormal behavior at baseline, were housed under standardized conditions. No additional specific inclusion or exclusion criteria were applied. APP/PS1 mice were selected because they represent a well-established model of Alzheimer’s disease (AD). These mice express a chimeric mouse/human amyloid precursor protein (Mo/HuAPP695swe) and a mutant human presenilin 1 (PS1-dE9), both associated with early-onset familial AD in humans. The humanized APP transgene enables the production of human Aβ peptides, which are central to amyloid plaque formation. This model was chosen because APP/PS1 mice reliably develop age-dependent beta-amyloid deposition in the brain, beginning at 6–7 months of age, thereby recapitulating key neuropathological features of human AD.

All animals were maintained at 22 ± 1 °C with a 12-h light/dark cycle and had free access to food and water. Mice were obtained from The Jackson Laboratory (Stock No. 005864), Bar Harbor, Maine, USA. All experimental procedures were conducted in accordance with Italian animal welfare regulations (D.M.116/92), the European Directive (2010/63/EU), and the ARRIVE guidelines. All efforts were made to minimize pain and distress, including the use of non-invasive oral treatment, enriched housing, and daily monitoring. No unexpected adverse effects related to PF-04691502 administration were observed. Humane endpoints were predefined (e.g., >20% weight loss, severe distress, seizures). Animals were monitored daily, and none required early euthanasia. The animal study protocol was approved by the Institutional Animal Care and Use Committee (IACUC) of the Ministero della Salute (protocol code 938/2024; approval date: 27 September 2024).

### 2.3. Treatment with PF-04691502

Mice were treated daily with PF-04691502 (HY-15177, MedChemExpress, Monmouth Junction, NJ, USA) (1 mg/kg) by oral administration for 12 weeks. The dose was consistent with previously published work [26]. PF-04691502 was administered orally at a dose of 1 mg/kg, corresponding to approximately one-tenth of the reported maximum tolerated dose in rodents (10 mg/kg) [26]. This dose selection was guided by pharmacokinetic data demonstrating rapid absorption (Cmax ~0.5 h), an apparent half-life of ~4.4 h and an oral bioavailability of ~63%. At 1 mg/kg, PF-04691502 ensures sufficient PI3K/mTOR target engagement while minimizing toxicity, as evidenced by the absence of body weight loss in treated animals (Supplementary Figure S1). The compound was formulated in 0.5% carboxymethylcellulose (CMC) dissolved in saline, according to the manufacturer’s instructions. At the end of the experimental period, the animals were sacrificed, and the whole brain was collected for downstream analyses.

### 2.4. Experimental Groups

Both APP/PS1 and WT mice were given PF-04691502 or vehicle daily for 12 weeks (*n* = 15/group). The number of mice per group was determined based on prior experience and data from the literature. Animals were randomly assigned to groups using the RAND() function in Microsoft Excel. To minimize potential confounders, the order of treatments was randomized daily, and all measurements were performed at the same time of day to reduce circadian-related variability. Additionally, cages were evenly distributed throughout the room, and their positions were rotated weekly to account for potential environmental effects related to cage location.

1. WT + vehicle: the animals in this group were vehicle-only-treated (15 males).
2. APP/PS1 + vehicle: the animals in this group were vehicle-only-treated (8 males and 7 females).
3. WT + PF: the animals in this group were treated with 1 mg/kg PF-04691502 (15 males).
4. APP/PS1 + PF: the animals in this group were treated with 1 mg/kg PF-04691502 (5 males and 10 females).

Group allocation was known only to the researcher responsible for assigning animals to treatment groups. The personnel conducting the experiments, performing outcome assessments, and analyzing the data were blinded to group allocation to minimize bias. All animals were included in the experiments and subsequent data analysis.

### 2.5. Behavioral Test

The Morris water maze (MWM) test was performed for 5 consecutive days to evaluate memory and spatial learning. The test was conducted as previously described [27]. Briefly, the test was conducted using a circular tank with a diameter of 1.5 m, located in a room with several visible cues on the walls. Inside the tank, there was a platform made invisible to the mice by the coloration of the water, which was kept at a constant temperature of 25 °C. The platform, measuring 14 cm in diameter, remained in a fixed position beneath the water’s surface at a depth of 1.5 cm. During training, the mice underwent four trials per day, starting from four pseudorandom entry points into the maze. If a mouse was unable to find the hidden platform within the 60-s time limit, the researcher gently guided it to the platform, where it was allowed to rest for 10 s. Between trials, a 25-s intertrial interval was maintained, during which each mouse was returned to its home cage. On the sixth day, the platform was removed, and the mice were permitted to swim freely in the pool for 60 s. During this probe trial, the latency to reach the former platform location, the number of crossings over that area, and the time spent in the target quadrant were analyzed manually, based on video recordings reviewed by trained observers blinded to the experimental groups. Data from the Morris Water Maze test were analyzed using PRISM software (version 9.5.1; SPSS Inc., Chicago, IL, USA).

### 2.6. Western Blot Analyses

Western blot analyses were conducted to assess mTOR levels, Microtubule-Associated Proteins 1A/1B Light Chain 3B (MAPLC3 or LC3), Beclin-1 (BECN1), Sequestosome 1/p62 (SQSTM1/p62), Lysosome-associated membrane protein 2 (LAMP-2) and phospho-p70 S6 Kinase (p-p70S6 kinase). After the sacrifice, the animal’s whole brains were removed and cytosolic and nuclear fractions were extracted, as described by Ardizzone et al. [28]. After SDS-PAGE, proteins were transferred from the polyacrylamide gel onto a PVDF membrane. Membranes were then blocked for 1 h at room temperature using 5% (*w*/*v*) nonfat dry milk in buffered saline (PM), followed by overnight incubation at 4 °C with the following primary antibodies: anti-phospho-mTOR (Ser2448) (1:500; Cell Signaling Technology #2972, Danvers, MA, USA), anti-MAPLC3/LC3 (1:500; Santa Cruz Biotechnology, sc-398822, Dallas, TX, USA), anti-BECN1 (1:500; Santa Cruz Biotechnology, sc-48381, Dallas, TX, USA), anti-LAMP-2 (1:500; Invitrogen #MA1-205, Waltham, MA, USA), anti-phospho-p70S6K (1:500; ABclonal #AP0564, Woburn, MA, USA), anti-AKT (1:500; Santa Cruz Biotechnology, sc-271966, Dallas, TX, USA) and anti-p-AKT (1:500; Santa Cruz Biotechnology, sc-48381, Dallas, TX, USA). Following incubation, membranes were thoroughly washed and then exposed to the appropriate secondary antibody (1:1000; Jackson ImmunoResearch, West Grove, PA, USA) for 1 h at room temperature. β-actin was used as the loading control (anti-β-actin, 1:500; sc-47778; Santa Cruz Biotechnology, Dallas, TX, USA). Protein signals were detected using an enhanced chemiluminescence (ECL) detection system, following the manufacturer’s instructions. Band intensities for each protein were quantified using ImageQuant TL software (v2003) and normalized to β-actin unless otherwise specified. Densitometric analysis was performed using Bio-Rad ChemiDoc™ software version 6.1 to determine relative expression levels.

### 2.7. Immunohistochemical Analysis of Tyrosine Hydrolase (TH) and β-Amyloid 1-42

Immunohistochemistry analysis of TH in the substantia nigra of mouse brains was conducted as described by Esposito and colleagues [29]. Seven μm tissue sections were incubated overnight with the following primary antibodies: anti-Tyrosine Hydroxylase (TH) (1:100; sc-25269, Santa Cruz Biotechnology, Santa Cruz, CA, USA) and anti-β-Amyloid 1-42 (1:100; AB5078P, Merck Millipore, Burlington, MA, USA). Following incubation, sections were gently rinsed with PBS and treated with the appropriate secondary antibody using the VECTASTAIN Universal Quick Kit, Peroxidase, R.T.U. (PK-7800; Vector Laboratories, Burlingame, CA, USA). Immunoreactivity was visualized using the chromogenic substrate 3,3′-Diaminobenzidine (DAB), which produces a brown precipitate. Counterstaining was performed using Nuclear Fast Red, while Nissl bodies were visualized with Cresyl Violet. For the graphical representation of densitometric analyses, the percentage of positively stained areas (brown signal) was quantified through computer-assisted color image analysis. Imaging was carried out using a Nikon Eclipse Ci-L microscope, and representative images are presented at 10× and 40× magnification.

### 2.8. Congo Red Staining

To highlight the presence of amyloid plaques, we conducted a Congo Red staining. After removing the excess paraffin, sections were progressively hydrated with a decreasing scale of alcohol and then processed using a Congo Red kit (04-210822; Bio-Optica, Milan, Italy), according to the manufacturer’s protocol. Then the slides were dehydrated with an increasing scale of alcohol, up to xylene, and mounted [30]. The figures are shown at a magnification of 10× and 40×.

### 2.9. Statistical Analysis

Experimental data are presented as mean ± standard deviation (SD), with *N* indicating the number of animals used in each group. Statistical analyses were performed using One-Way or Two-Way ANOVA, followed by Bonferroni or Tukey’s post hoc tests for multiple comparisons, using GraphPad Prism version 8.0 (La Jolla, CA, USA). Immunofluorescence (IF) data were analyzed using the *t*-test. A *p*-value of less than 0.05 was considered statistically significant.

## 3. Results

### 3.1. PF-04691502 Treatment Improves Spatial Learning and Memory in APP/PS1 Mice

PF-04691502 administration did not induce relevant alterations in body weight during the intervention. As shown in the Supplementary Figure S1, body weight profiles of treated mice overlapped with those of respective controls, indicating good tolerability of the treatment.

**Figure 1:**
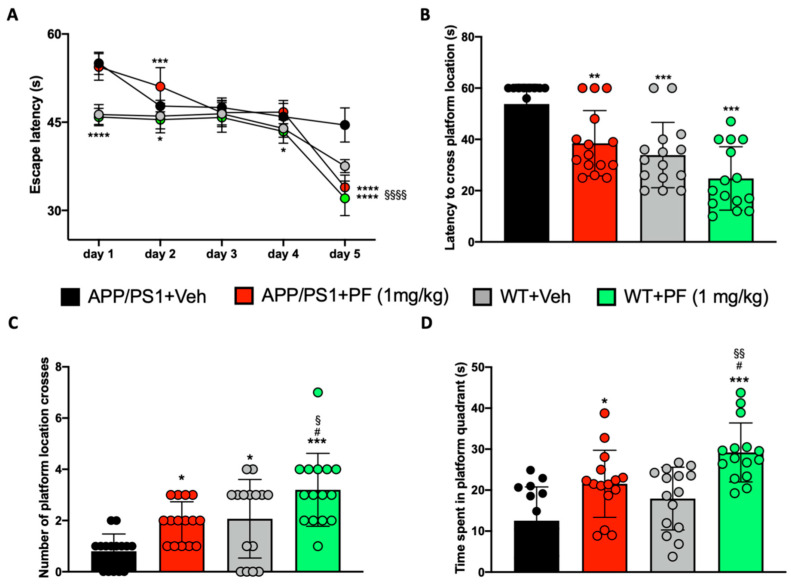
Effect of PF-04691502 treatment on learning and memory. PF-04691502 improved learning in APP/PS1 mice compared to the APP/PS1 + Vehicle group (**A**). APP/PS1 mice treated with PF-04691502 took less time to cross the platform than those administered vehicle only (**B**). APP/PS1 + PF-treated animals showed an increase in time spent in the quadrant where the platform used to be (**D**) and in the number of platform location crosses (**C**), compared to the APP/PS1 + Vehicle group. The WT + PF-treated group exhibited the most significant behavioral improvement compared to the WT and APP/PS1 control groups. §/*/# *p* < 0.05 vs. WT + Veh/APP/PS1 + Veh/APP/PS1 + PF; §§/** *p* < 0.01 vs. WT + Veh/APP/PS1; *** *p* < 0.001 vs. APP/PS1 + Veh; §§§§/**** *p* < 0.0001 vs. WT + Veh/APP/PS1 + Veh.

To assess the effects of PF-04691502, we tested spatial learning and memory in treated mice using the Morris water maze (MWM). Notably, our study was not powered to identify potential sex differences. On the first day of testing, no significant differences were observed among groups (Supplementary Figure S2), highlighting the absence of major motor impairments at baseline. We found that PF-04691502 treatment for 12 weeks had a significant effect on learning (two-way ANOVA, *p* < 0.0001; Figure 1A).

The Bonferroni multiple comparisons test revealed that the APP/PS1 + PF group showed significantly improved performance compared to the APP/PS1 + vehicle group on day 2 (*p* < 0.001) and day 5 (*p* < 0.0001). Additionally, wild-type (WT) mice treated with PF outperformed the APP/PS1 + vehicle group on days 1 (*p* < 0.0001), 2 (*p* < 0.05), 4 (*p* < 0.05), and 5 (*p* < 0.0001). On day 5, PF-treated WT mice also performed significantly better than the WT + vehicle group (*p* < 0.0001). Notably, no statistically significant differences in this behavior were observed between male and female mice within the APP/PS1 vehicle and APP/PS1 PF-04691502-treated groups as shown in Supplementary Figure S3A.

To test reference memory, 24 h after the last training trial, we removed the hidden platform and gave the mice an additional 60-s training trial. We recorded the amount of time taken to cross the location where the platform was hidden (Figure 1B), the number of times each animal crossed the platform location (Figure 1C), and the time spent in the quadrant where the platform was originally located (Figure 1D). PF-04691502 affected reference memory in all three measurements, as indicated by one-way ANOVA analysis (*p* < 0.001). Bonferroni correction indicated that WT + PF-treated mice performed significantly better than APP/PS1 + vehicle mice (*p* < 0.001) in all aspects analyzed (Figure 1B–D) and better than the WT + vehicle group (*p* < 0.05, Figure 1C,D). Additionally, we observed a significant improvement in reference memory in the APP/PS1 mice treated with PF-04691502 compared to the APP/PS1 animals that received the vehicle. Specifically, APP/PS1 + PF-treated mice showed a shorter time to the first platform location cross (*p* < 0.01), a higher number of platform location crosses (*p* < 0.05), and spent more time in the target quadrant (*p* < 0.05) than the APP/PS1+ vehicle mice (Figure 1B–D). Gender-based analysis revealed that female APP/PS1 mice showed significantly better performance than males in terms of the number of platform crossings and time spent in the target quadrant as shown in Supplementary Figure S3C,D. Comparative analysis revealed no statistically significant sex-related differences in the latency to the first platform location crossing among APP/PS1 vehicle and APP/PS1 PF-04691502-treated groups as shown in Supplementary Figure S3B. Taken together, these data indicate the positive effects of PF-04691502 on spatial learning and memory. Further studies are needed to determine whether PF-04691502 affects other cognitive domains in these mice.

### 3.2. PF-04691502 Treatment Reduces Hyperactivity of mTOR and p-p70S6K

**Figure 2:**
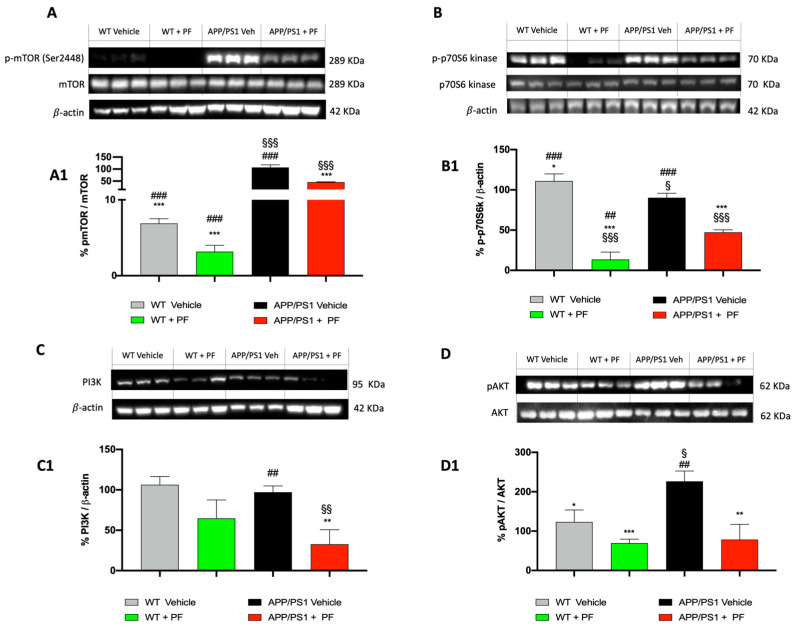
Inhibition of the active form of mTOR and p-p70S6K. PF-04691502 reduced the levels of p-mTOR in treated APP/PS1 mice compared to those that received vehicle only (**A**,**A1**). The levels of p-p70S6K were also reduced in both WT and APP/PS1 animals treated with PF-04691502 compared with vehicle-treated animals (**B**,**B1**). PF-04691502 reduced the levels of PI3K (**C**,**C1**) and pAKT (**D**,**D1**) in treated APP/PS1 mice compared to those that received vehicle only. Results were normalized using mTOR and β-actin as controls for p-mTOR, p-p70S6 kinase, PI3K, respectively. Data are expressed as mean ± SD. In each experimental group, the number of mice was *n* = 15. §/* *p* < 0.05 vs. WT + Veh/APP/PS1 + Veh; §§/**/## *p* < 0.01 vs. WT + Veh/APP/PS1 + Veh/APP/PS1 + PF; §§§/***/### *p* < 0.001 vs. WT + Veh/APP/PS1 + Veh/APP/PS1 + PF.

Several laboratories have reported that the PI3K/mTOR signaling pathway is overactive in AD and is associated with the accumulation of Aβ and Tau [31,32,33,34,35]. To assess the changes in mTOR signaling following PF-04691502 treatment, we measured levels of phosphorylated mTOR (p-mTOR Ser2448) and p70S6 kinase (p-p70S6). Analysis by one-way ANOVA indicated that p-mTOR levels differed among the groups (Figure 2A, score A1; *p* < 0.001). Bonferroni correction showed that p-mTOR levels were significantly higher in APP/PS1 mice receiving vehicle compared to WT mice also treated with vehicle (*p* < 0.001; Figure 2A, score A1), which is consistent with reports from the literature indicating that mTOR signaling is overactive in APP/PS1 mice [36]. Notably, PF-04691502 treatment significantly reduced the p-mTOR (Ser2448) levels in APP/PS1 mice compared to APP/PS1 mice administered vehicle only (Figure 2A, *p* < 0.001). Similarly, PF-04691502 also altered p-p70S6 levels (Figure 2B, score B1; one-way ANOVA *p* < 0.001) and PI3K levels (Figure 2C, score C1; one-way ANOVA *p* < 0.01). Post hoc analyses indicated that the APP/PS1 + PF-treated group was significantly different from the APP/PS1 + vehicle group (*p* < 0.001). PF-04691502 administration also significantly reduced p-p70S6 levels in WT animals compared to the WT + Vehicle group (Figure 2B, score B1; *p* < 0.001). In addition, our data showed that PF-04691502 treatment led to a reduction in p-Akt levels in APP/PS1 group (Figure 2D, score D1; *p* < 0.01), consistent with the reported capacity of this compound to inhibit mTORC2 signaling. These findings confirm that, in addition to targeting mTORC1, PF-04691502 also impacts the mTORC2–Akt axis in our system, which may contribute to its overall effects.

### 3.3. PF-04691502 Treatment Reduces Aβ Accumulation

**Figure 3:**
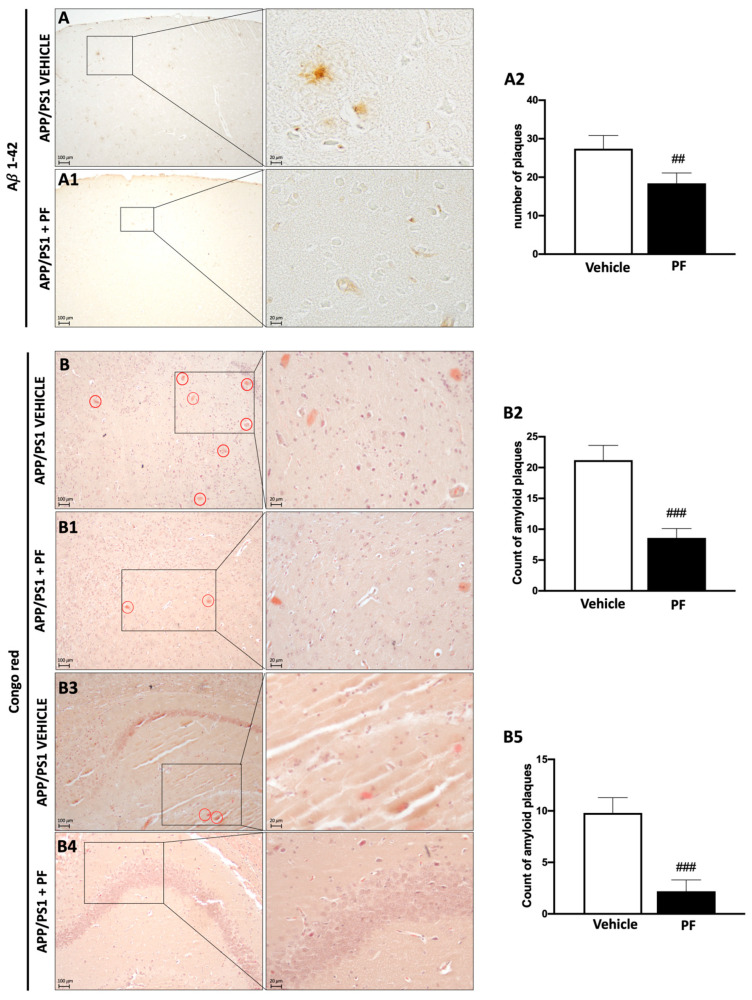
Histological evaluation of Aβ plaque positivity in brain tissue of APP/PS1 transgenic mice. Treatment with PF-04691502 decreased the accumulation of Aβ compared to animals treated only with vehicle (**A**–**A2**). The group of APP/PS1 mice treated with PF-04691502 also exhibited a reduced number of beta-amyloid plaques in the cortex (**B**–**B2**) and hippocampus (**B3**–**B5**) as observed by Congo Red staining, compared to the APP/PS1 + vehicle group. Figures are shown at magnifications of 20× and 40×. Data are expressed as mean ± SD. Each experimental group consisted of *n* = 15 mice. Unpaired *t*-test; ## *p* < 0.01 vs. APP/PS1 + PF; ### *p* < 0.001 vs. APP/PS1 + PF.

To assess the efficacy of the treatment on AD-like neuropathology, we first evaluated Aβ pathology by immunohistochemical analysis. We found that Aβ1-42 was significantly reduced in APP/PS1 animals treated with PF-04691502 compared to APP/PS1 animals that received vehicle only (Figure 3(A–A2)). To further assess Aβ pathology, we used Congo red staining, which highlights mature Aβ plaques [37]. This analysis showed a significant accumulation of Aβ plaques in the cortex (Figure 3(B–B2)) and hippocampus (Figure 3(B3–B5)) of APP/PS1 animals administered vehicle only. PF-04691502 treatment for 12 weeks markedly reduced Aβ plaques in the APP/PS1 animals. In addition, we also observed that PF-04691502 significantly reduced total Tau expression compared to APP/PS1 animals treated with vehicle, as demonstrated by Western blot analysis using the T46 antibody (see Supplementary Figure S4).

### 3.4. PF-04691502 Modulates Autophagy

**Figure 4:**
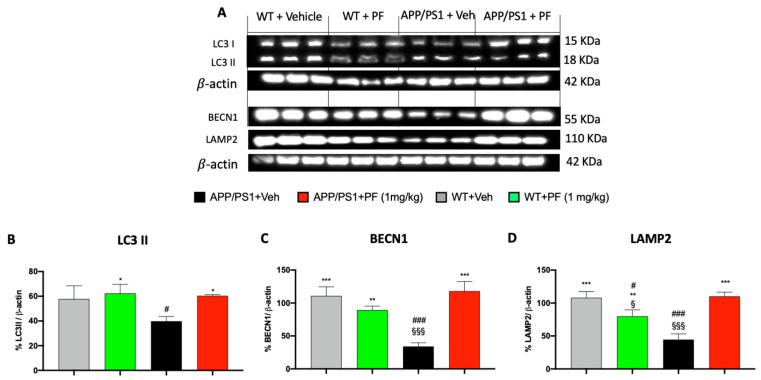
Effects of PF-04691502 on autophagy. (**A**) Western blot representation of cytosolic brain fraction analysis in WT and APP/PS1 mice. We observed an increase in the levels of LC3II (**B**) and BCN1 (**C**) in the APP/PS1 + PF-treated group compared to the APP/PS1 + vehicle group. The APP/PS1 + vehicle group showed decreased levels of LAMP-2 (**D**), which were increased by treatment with PF-04691502. Data are expressed as mean ± SD. Each experimental group consisted of *n* = 15 mice. §/*/# *p* < 0.05 vs. WT + Veh/APP/PS1 + Veh/APP/PS1 + PF; ** *p* < 0.01 vs. APP/PS1 + Veh; §§§/***/### *p* < 0.001 vs. WT + Veh/APP/PS1 + Veh/APP/PS1 + PF.

mTOR is a negative regulator of autophagy [38,39,40]. Therefore, we hypothesized that a decrease in mTOR signaling would increase autophagy induction. To assess possible PF-04691502-mediated changes in autophagy, we evaluated several autophagy markers by Western blot. Beclin-1 (BECN1) is a well-known autophagy inducer that regulates the localization of other autophagy-related proteins in autophagosomes [41]. Overexpression of BECN1 and the consequent activation of autophagy can prevent neuronal cell death and promote the elimination of toxic protein aggregates [42]. In fact, some studies show that a significant decrease in BECN1 levels reduces neuronal autophagy, ultimately contributing to neurodegeneration [41,43]. Microtubule-associated protein 1A/1B-light chain 3 (LC3) is another autophagy-specific marker and consists of two key subtypes: LC3I and LC3II. The transformation from LC3I to LC3II is associated with the activation of autophagy. We found that 12-week treatment with PF-04691502 changed the levels of LC3II and BECN1 (Figure 4A–C; one-way ANOVA, *p* < 0.001). Bonferroni correction indicated that the levels of both proteins were significantly higher in the APP/PS1 + PF-treated group compared to the APP/PS1 + Veh group (LC3II *p*< 0.01; BCN1 *p* < 0.001; Figure 4A–C). Conversely, in the WT groups, BCN1 levels remained unchanged, indicating that PF treatment did not affect basal autophagy marker expression in non-pathological conditions.

LAMP-2 is a lysosomal protein required for the final phase of autophagy, which involves the fusion of autophagosomes with lysosomes [44,45]. In addition, LAMP-2 is known as a receptor present in the lysosomal membrane for the substrate chaperone-mediated autophagy proteins [46] and is functionally involved in cholesterol export from late endosomes and lysosomes [47,48]. Both processes are critical for proper lysosomal function and have been shown to be significantly disrupted in various neurodegenerative disorders, including Alzheimer’s disease (AD) [49]. To further investigate the impact of PF-04691502 on autophagy, we assessed LAMP-2 expression levels and observed significant differences across the four experimental groups (Figure 4A,D; one-way ANOVA, *p* < 0.001). Post hoc analysis using the Bonferroni correction revealed that LAMP-2 levels were significantly elevated in APP/PS1 mice treated with PF-04691502 compared to those receiving vehicle treatment (*p* < 0.001; Figure 4A,D).

### 3.5. PF-04691502 Treatment Increases the Expression of TH

**Figure 5:**
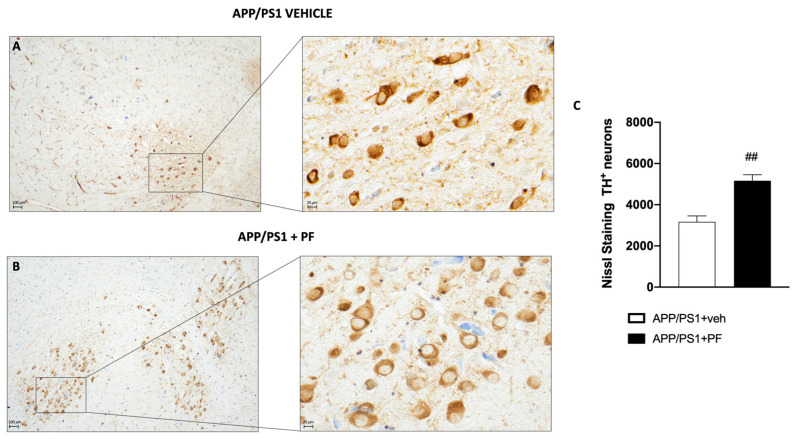
Histological evaluation of TH positivity in the substantia nigra of APP/PS1 transgenic mice. Treatment with PF-04691502 increased the immunopositivity staining of TH ((**B**), score (**C**)), compared with animals treated only with the vehicle (**A**). Figures are shown at a magnification of 20×. Data are expressed as mean ± SD. In every experimental group, the number of mice was *n* = 15. Unpaired *t*-test. ## *p* < 0.01 vs. APP/PS1 + PF.

Dopamine plays an important role in learning and memory and deficits in this neurotransmitter are linked to AD [50]. Notably, the literature presents conflicting evidence about the mechanistic link between mTOR signaling and dopaminergic function [51,52,53]. To determine the effect of PF-04691502 on dopaminergic neurons in APP/PS1 mice, we evaluated the number of tyrosine hydroxylase (TH) positive neurons by immunohistochemistry. We found that PF-04691502 significantly increased TH levels in the APP/PS1 + PF-treated mice compared to APP/PS1 + vehicle mice (Figure 5).

### 3.6. PF-04691502 Attenuates Glial Activation and Neuroinflammation

**Figure 6:**
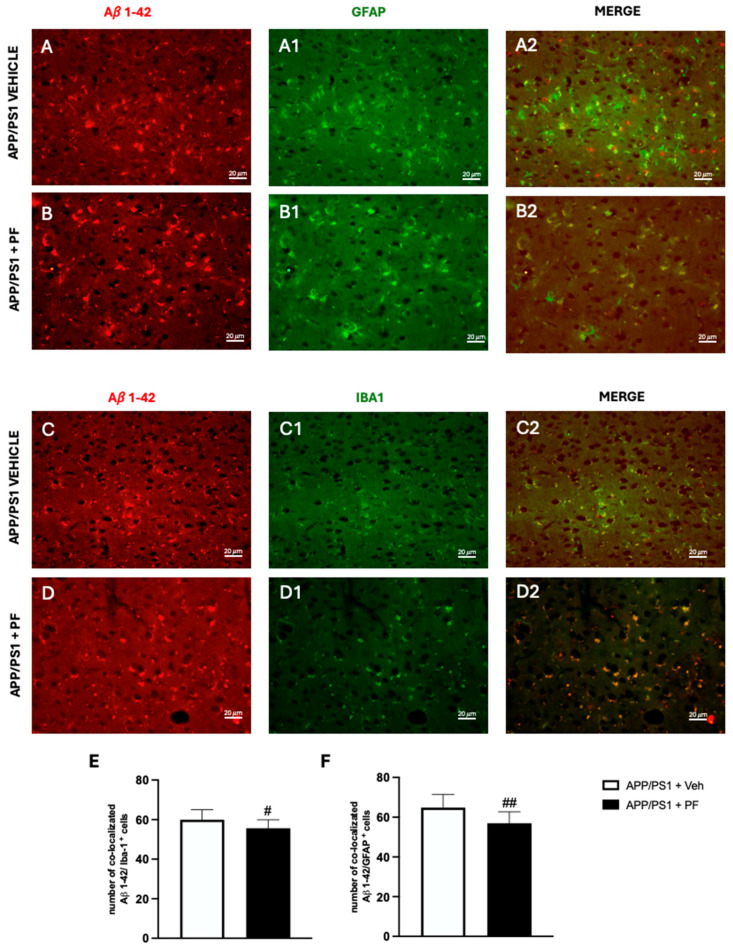
PF-04691502 reduces astrocytic and microglial activation in APP/PS1 mice. Representative immunofluorescence images showing GFAP (astrocytes, green (**A1**,**B1**)), Iba1 (microglia, green (**C1**,**D1**)) immunoreactivity co-labeled with Aβ1-42 (red, (**A**–**D**)) in cortical regions of APP/PS1 + Veh, and PF-04691502-treated APP/PS1 mice. The merged images of GFAP with Aβ are shown in (**A2**,**B2**), while the merged images of Iba1 with Aβ are presented in (**C2**,**D2**). Quantification of GFAP (**E**) and Iba1 (**F**) signal intensity. Data are expressed as mean ± SD. Each experimental group consisted of *n* = 15 mice (# *p* < 0.05, ## *p* < 0.01). Scale bar: 40×, 20 μm.

Immunofluorescence analysis revealed a marked increase in the activation of both astrocytes and microglia in the APP/PS1 group, as indicated by enhanced GFAP and Iba1 immunoreactivity (Figure 6A–C). Interestingly, this glial activation appeared to be attenuated in the PF-04691502-treated animals, with a noticeable reduction in both astrocytic and microglial reactivity compared to untreated APP/PS1 mice (Figure 6B–D). These findings suggest that PF-04691502 treatment not only impacts Aβ burden but may also modulate the neuroinflammatory response by dampening glial activation. 

## 4. Discussion

Strong evidence links the PI3K/mTOR pathway to AD pathogenesis. In previous studies, we have shown that mTOR is hyperactive in human brains and in transgenic mice of AD brains, demonstrating the close correlation between mTOR and the progression of Aβ and Tau [35,36]. In addition, inhibition of this signaling pathway leads to improvements in lifespan and health span in both wild-type mice and several mouse models of human diseases. Based on these, decreasing the PI3K/mTOR signaling could be a suitable strategy for treating AD. Here, we tested PF-04691502, a dual PI3K/mTOR inhibitor, for 12 weeks in a murine model of AD.

During the MWM test, both WT and APP/PS1 animals that received PF-04691502 orally showed an improvement in learning and memory throughout the test. PF-04691502 treatment in APP/PS1 animals yielded promising results; indeed, the animals already after a few days began to reach the platform hidden under the surface of the water proving to have learned the position and being able to orient in the space available. These results are consistent with data in the literature linking mTOR hyperactivation with memory deficit in mouse models of AD [54,55]. For example, excessive hippocampal mTOR signaling leads to impairments in learning and memory in an animal model of tuberous sclerosis [56]. However, these deficits can be alleviated by administering rapamycin, an mTOR inhibitor. Additionally, rapamycin has been shown to ameliorate memory impairments associated with cannabinoid consumption and with the buildup of Aβ and Tau [35,57,58]. Notably, our results indicate that inhibition of mTOR/PI3K enhances learning and memory even in WT mice, suggesting the possibility of a protective role in increasing both health span and lifespan independent from pathology. This is consistent with previous results indicating that chronic rapamycin administration ameliorates age-dependent cognitive decline in wild-type mice [59].

To confirm the action on the target, we evaluated the molecular expression of some key factors of the PI3K/mTOR pathway in the brains of APP/PS1 mice treated with the inhibitor. Treatment with PF-04691502 reduced the levels of p-mTOR, confirming the modulation of this signaling pathway, often linked to the improvement of learning and memory [56,60]. One of the most significant targets of this complex is p70S6K, which is activated by mTOR-dependent phosphorylation of threonine 389 (T389). Once phosphorylated, p-p70S6K inhibits the initiation of protein synthesis [61]. Our data also suggest that this protein is inhibited by 12 weeks of treatment with PF-04691502, confirming that it modulates key proteins of the PI3K/mTOR pathway.

To assess Aβ load in 18-month-old mice, we performed different histological analyses; transgenic animals treated with the vehicle showed numerous Aβ plaques, which were reduced by chronic PF-04691502 administration. This result was also confirmed by Congo Red staining, which revealed a decreased immunopositive signal of Aβ, especially in the cortex and hippocampus. Our findings align with prior research indicating that rapamycin treatment decreases the accumulation of Aβ and fibrillar aggregates of Aβ by approximately 40–50% in 3xTg-AD mice over 16 months [58]. Similarly, Galvan’s team reported a reduction in Aβ levels in the brains of hAPP(J20) mice within a few months of rapamycin treatment [55], along with a decrease in amyloid plaques during later stages of AD-like disease [62]. The effects of rapamycin on AD-like pathology have also been studied in APP/PS1 mice. Consistent with the data reported here, inhibition of mTOR with rapamycin decreased Aβ deposition, enhanced synaptic plasticity, and improved learning and memory. These changes were associated with increased autophagy induction [63,64].

Unlike rapamycin, which selectively inhibits mTORC1, PF-04691502 is a dual inhibitor of mTORC1/2, potentially leading to broader effects on cellular metabolism and survival pathways. Everolimus and temsirolimus, rapamycin analogues and selective mTORC1 inhibitors, have also been studied for their potential neuroprotective effects in AD, due to their ability to enhance autophagy, reduce Aβ accumulation, and alleviate tau pathology [65,66]. However, their selective inhibition of mTORC1 may have limitations, as mTORC2 activity remains intact, potentially triggering compensatory feedback mechanisms that support AKT signaling and diminish the overall efficacy of autophagy induction. In contrast, PF-04691502 offers more comprehensive suppression of the mTOR pathway, resulting in a more sustained autophagic response [67]. Indeed, PF-04691502, as a dual PI3K/mTOR inhibitor, was shown to suppress both mTORC1 and mTORC2, attenuating AKT signaling and thereby avoiding the compensatory feedback loops associated with selective mTORC1 inhibition [25].

In recent years, accumulating evidence has demonstrated a link between AD-related memory dysfunction and deficits in dopaminergic signaling in patients [68,69,70] and in several experimental models of AD [50,71,72,73]. Conflicting evidence is present in the literature as the mechanistic link between mTOR signaling and dopaminergic function [51,52,53]. For example, Kosillo and colleagues showed that increasing mTOR activity in dopaminergic neurons alters synaptic structure and impairs dopamine release in the striatum [51]. In contrast, Santini and colleagues showed that inhibition of mTOR improves overall dopaminergic function [53]. Here, we evaluated TH levels in the brains of APP/PS1 mice. Our results showed that the treatment of 12 weeks with PF-04691502 increased the number of TH-positive cells, suggesting that decreasing mTOR signaling may restore dopaminergic function in APP/PS1 mice.

The PI3K/mTOR signaling is linked with the modulation of autophagy, a process of degradation and recycling of cellular components, including damaged cellular organelles, poorly folded proteins, and other unnecessary cellular structures [74]. Strong evidence suggests that autophagy plays a key role in the degradation of damaged Aβ and Tau proteins in the brain. In turn, when autophagy is impaired, due to aging or other factors, cells may have difficulty removing these abnormal proteins, which may accumulate in the brain and contribute to AD development [75,76]. The molecular analysis performed during this study showed that treatment with PF-04691502 significantly increased autophagy markers such as LAMP-2, LC3I/LC3II, and BECN1. This finding is consistent with observations suggesting that reduced autophagy activity is associated with age-related cognitive decline and AD pathology and its activation is required for memory formation [77,78,79]. In contrast, reducing mTOR signaling increases autophagy, which in turn leads to a reduction in Aβ plaques and an improvement in overall cognitive health [33].

Our data also suggest that autophagy induction may be impaired in untreated APP/PS1 mice. This finding is consistent with previous studies showing that markers such as p-mTOR, Beclin-1, and LAMP-2 are significantly altered in APP/PS1 transgenic mice compared to wild-type mice [42,80]. Contradicting results have been published on the LAMP-2 levels in APP/PS1 mice: while some studies report an increase in LAMP-2 expression, others have observed a decrease, which is consistent with our findings [80,81,82,83]. In fact our results support the LAMP-2 levels are decreased in APP/PS1 mice compared to age-matched control mice. These observations support the broader hypothesis that impaired autophagy induction may contribute to the development of Alzheimer’s disease [84]. In AD, the observed increase in glial activation may contribute to impaired autophagy, as pro-inflammatory mediators released by reactive astrocytes and microglia can disrupt lysosomal function and autophagic flux. By attenuating glial reactivity, PF-04691502 may help restore a more balanced autophagic process, limiting the detrimental pro-inflammatory responses typically associated with the M1-like phenotype while potentially preserving or enhancing the protective Aβ-clearing functions linked to M2-like microglial states [85,86]. This modulation could contribute to the observed reduction in Aβ and Tau pathology, supporting the notion that regulating glial activation is a crucial component of effective AD therapeutics.

We acknowledge the limitations of our study related to the lack of sex-based analyses. Our experimental design was not originally conceived to assess sex as a biological variable, and therefore was not powered or structured to systematically evaluate sex-related differences. However, we recognize the scientific relevance of this aspect, particularly in the context of Alzheimer’s disease, where sex-specific differences in pathology and behavior have been previously reported.

To address this limitation as thoroughly as possible within the constraints of our study, we have included in the Supplementary Figure S3 a comparative analysis between male and female mice within the APP/PS1 vehicle and APP/PS1 PF-04691502-treated groups. This analysis was limited to these two groups, as WT mice in our study were exclusively male and therefore not suitable for a meaningful comparison between sexes. While these additional data are exploratory in nature and not part of the original study objective, we believe they provide useful insights and help to contextualize our findings. Nonetheless, the overall data suggest that PF-04691502 remains a promising candidate for the treatment of Alzheimer’s disease and other age-related disorders, independently of sex.

Another limitation of our study is the lack of direct evidence regarding PF-04691502 brain penetration, drug levels, and target engagement in central nervous system (CNS) tissues. Although the compound possesses favorable physicochemical characteristics, including moderate lipophilicity, good oral bioavailability, and a relatively low molecular weight (425.5 g/mol), no published preclinical studies have quantitatively assessed its distribution or the extent of sustained target inhibition specifically in the brain. While these properties support the possibility of blood–brain barrier permeability, this remains to be experimentally validated.

Notably, PF-04691502 was originally developed as an anticancer drug and underwent a phase 1 clinical trial, where the maximum tolerated dose (MTD) was defined at 8 mg per day. Dose-limiting toxicities included grade 3 fatigue at 8 mg, grade 3 rash at 11 mg, and grade 2 intolerable fatigue at 11 mg. Common treatment-related adverse events comprised fatigue, decreased appetite, nausea, hyperglycemia, rash, and vomiting. The observed alteration in glucose homeostasis, characterized by increased fasting glucose, insulin, and C-peptide levels, indicates a significant systemic metabolic impact [25]. Importantly, some of these side effects, such as fatigue and decreased appetite, could also reflect central nervous system involvement, providing indirect clinical evidence supporting the hypothesis that PF-04691502 may cross the blood–brain barrier. Importantly, the behavioral improvements observed in our APP/PS1 mice following PF-04691502 treatment suggest functional CNS effects, indirectly supporting the compound’s activity within the brain. Taken together, these preclinical and clinical observations suggest a potential for CNS penetration and activity of PF-04691502, although further studies are needed to directly measure brain drug levels and confirm target engagement within CNS tissues.

## 5. Conclusions

The results shown here suggest that changes in the autophagy pathway following mTOR inhibition improve learning and memory. Thus, although PF-04691502 deserves further investigation to clarify its efficacy and safety, it can be considered a valid therapeutic agent, capable of improving learning and memory while decreasing Aβ deposits in the brain.

## Data Availability

The original contributions presented in this study are included in the article/Supplementary Materials. Further inquiries can be directed to the corresponding author.

## Supplementary Materials

The following supporting information can be downloaded at: https://www.mdpi.com/article/10.3390/cells14181474/s1, Figure S1. Body weight of WT and APP/PS1 mice during the 12-week treatment period with PF- 04691502 or vehicle. No statistically significant differences were observed between treated and respective control groups in either genotype over time. Data are presented as mean ± SD. Figure S2. Day 1 performance of MWM. Behavioral assessment on the first day of testing revealed no statistically significant differences in learning among groups, indicating the absence of major motor impairments at baseline. Data are presented as mean ± SD. Figure S3: Gender differences in the effects of PF treatment on spatial learning and memory in APP/PS1 mice. (A) Escape latency during the 5-day training phase of the Morris Water Maze (MWM). (B) Latency to cross the previous platform location during the probe trial. (C) Number of platform location crosses during the probe trial. (D) Time spent in the target quadrant during the probe trial. Data are shown as mean ± SD. * vs. APP/PS1 Vehicle (male); ° vs. APP/PS1 Vehicle (female); # vs. APP/PS1 + PF (male); + vs. APP/PS1 + PF (female). */°/#/+ *p* < 0.05;**/°°/##/++ *p* < 0.01; ****/°°°°/####/++++ *p* < 0.0001. Figure S4: Effects of PF-04691502 on Tau expression. Western blot representation of cytosolic brain fraction analysis in WT and APP/PS1 mice. We observed a decrease in the levels of T46 in the APP/PS1+PF treated group compared to the APP/PS1+vehicle group. Data are expressed as mean ± SD. Each experimental group consisted of n = 15 mice. §§§/***/### *p* < 0.001 vs. WT+Veh/APP/PS1+Veh/APP/PS1+PF.

## Abbreviations

The following abbreviations are used in this manuscript:

AD	Alzheimer’s disease
Aβ	Amyloid-β
APP	Amyloid precursor protein
PS1	Presenilin 1
PS2	Presenilin 2
PI3K	Phosphatidylinositol 3-kinase
mTOR	Mammalian target of rapamycin
Akt	Protein kinase B
WT	Wild-type
MWM	Morris water maze
MAPLC3	Microtubule-Associated Proteins 1A/1B Light Chain 3B
BECN1	Beclin-1
SQSTM1/p62	Sequestosome 1/p62
LAMP-2	Lysosome-associated membrane protein
p-p70S6 kinase	Phospho-p70 S6 Kinase
TH	Tyrosine Hydrolase
