# Supplementary material for: PF-04691502, a PI3K/mTOR Dual Inhibitor, Ameliorates AD-like Pathology in a Mouse Model of AD
Source: Cells. 2025 Sep 21;14(18):1474. doi: 10.3390/cells14181474 (PMC12468398; doi:10.3390/cells14181474)
Supplement: Supplementary file 1 [file cells-14-01474-s001.zip › cells-3749784-supplementary.pdf]

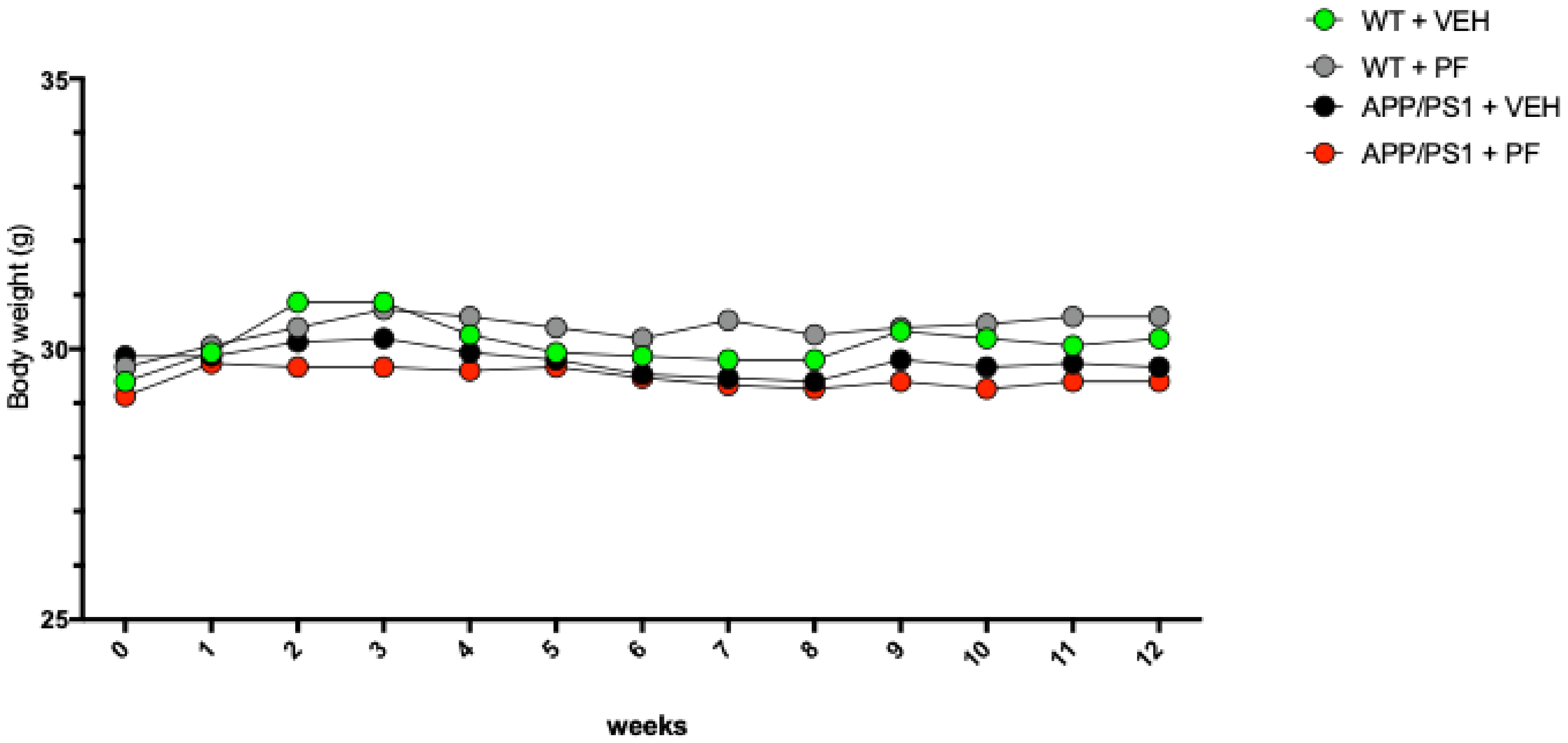

Supplementary Figure S1. Body weight of WT and APP/PS1 mice during the 12-week treatment period with PF-04691502 or vehicle. No statistically significant differences were observed between treated and respective control groups in either genotype over time. Data are presented as mean ± SD.

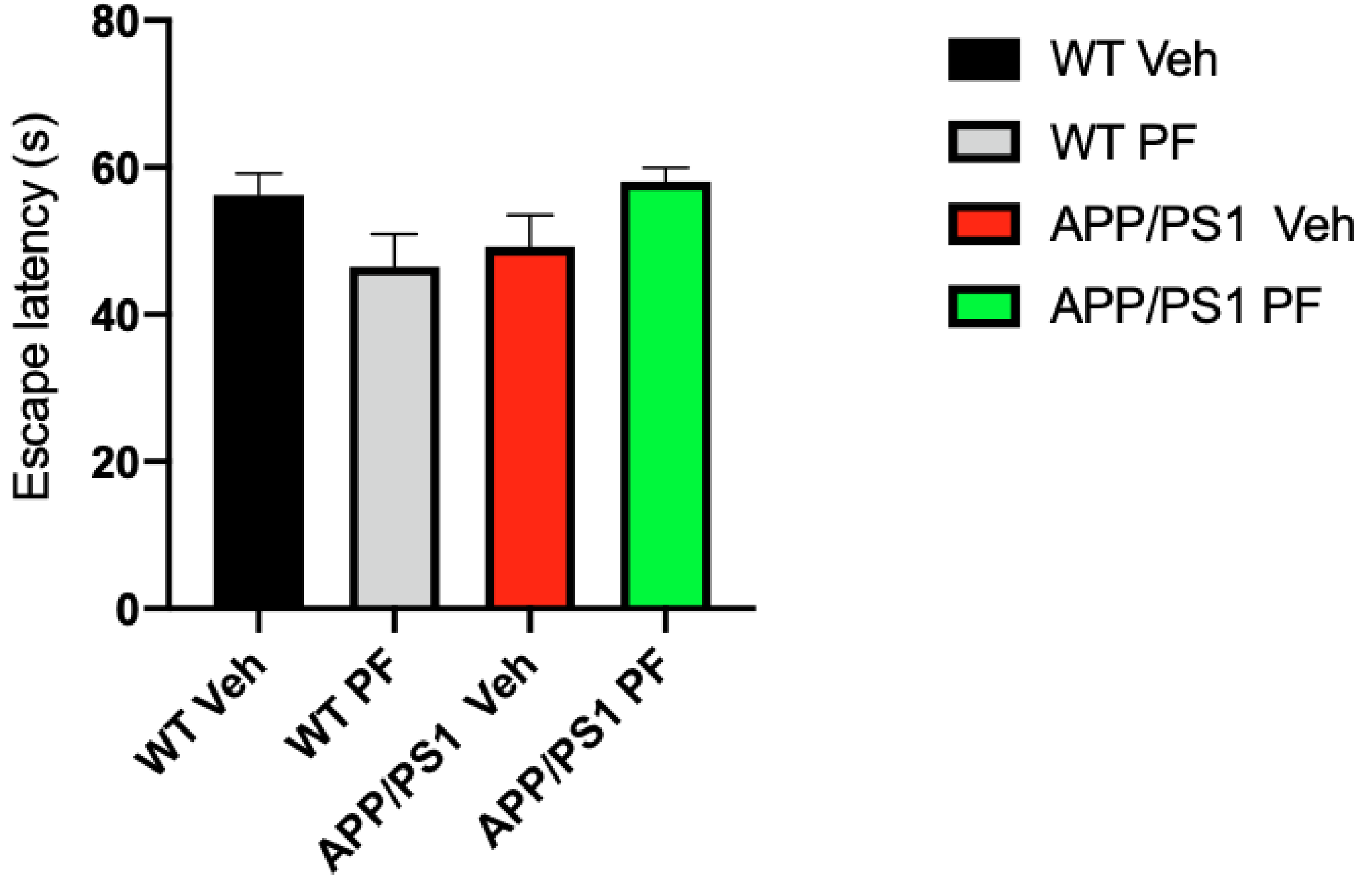

Supplementary Figure S2: Day 1 performance of MWM. Behavioral assessment on the first day of testing revealed no statistically significant differences in learning among groups, indicating the absence of major motor impairments at baseline. Data are presented as mean ± SD.

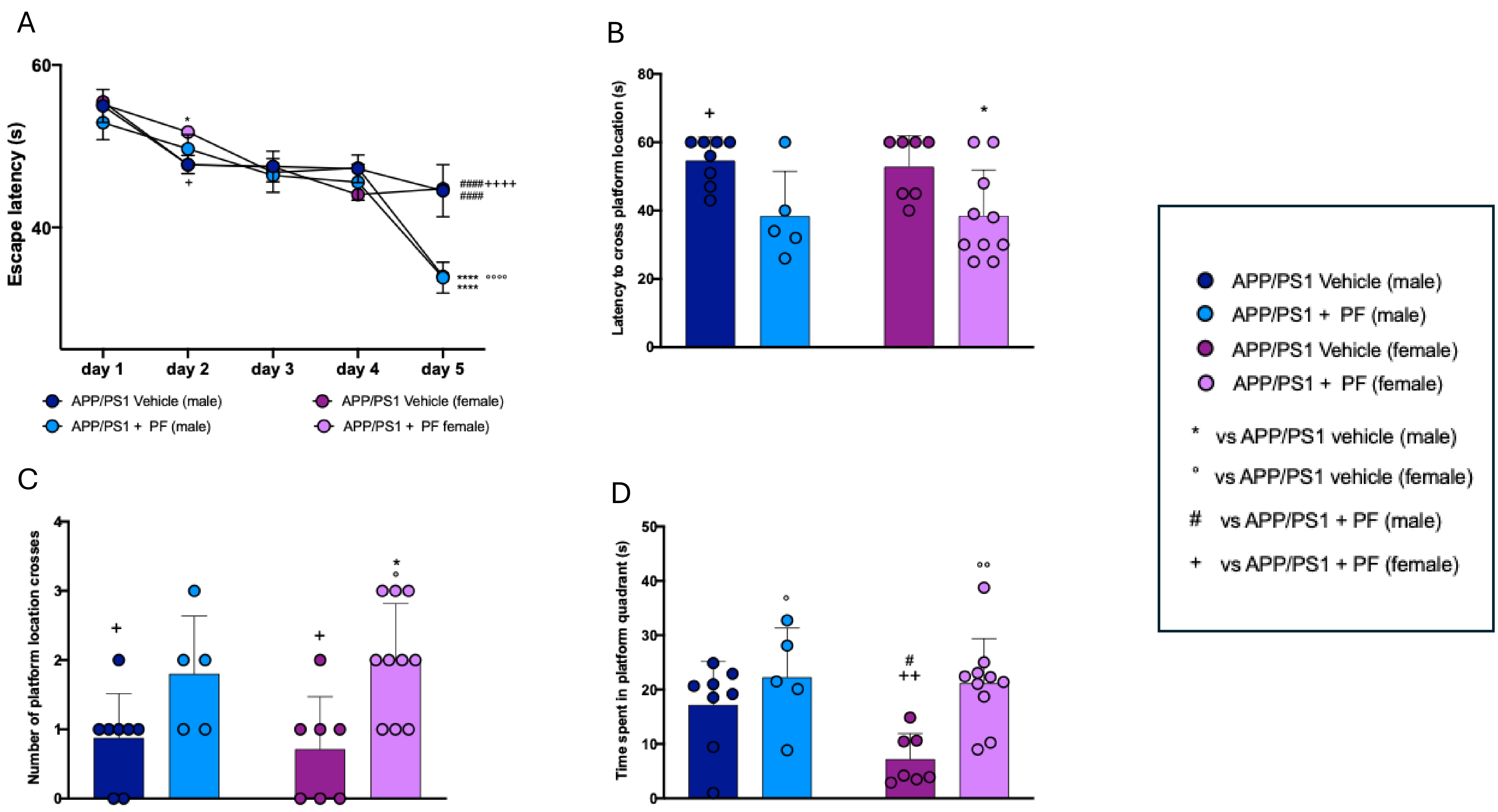

Supplementary Figure S3: Gender differences in the effects of PF treatment on spatial learning and memory in APP/PS1 mice. (A) Escape latency during the 5-day training phase of the Morris Water Maze (MWM). (B) Latency to cross the previous platform location during the probe trial. (C) Number of platform location crosses during the probe trial. (D) Time spent in the target quadrant during the probe trial. Data are shown as mean ± SD. * vs. APP/PS1 Vehicle (male); ° vs. APP/PS1 Vehicle (female); # vs. APP/PS1 + PF (male); + vs. APP/PS1 + PF (female). */°/#/+ $p < 0.05$; **/°°/##/++ $p < 0.01$; ****/°°°°/####/++++ $p < 0.0001$

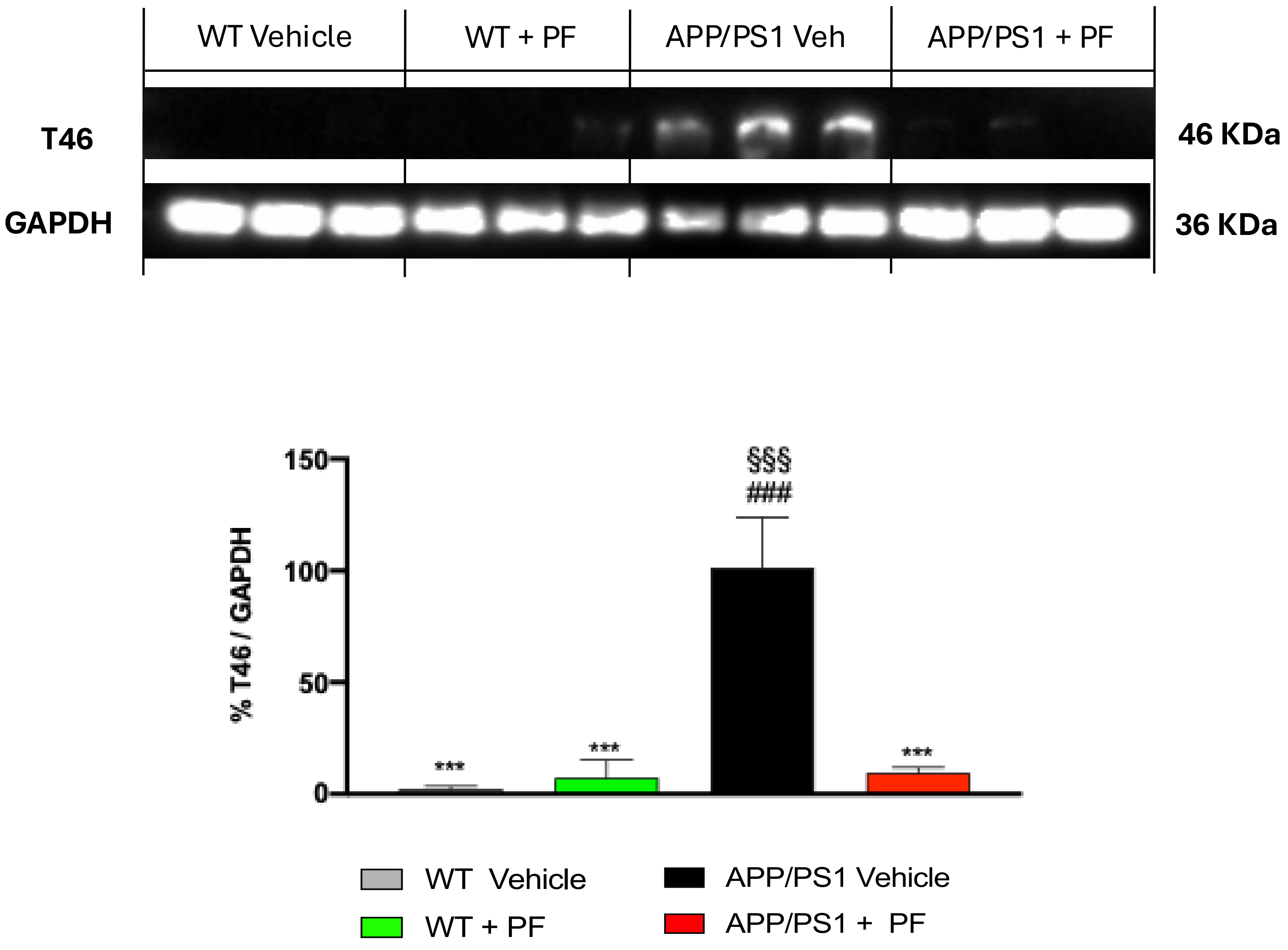

Supplementary Figure S4: Effects of PF-04691502 on Tau expression. Western blot representation of cytosolic brain fraction analysis in WT and APP/PS1 mice. We observed a decrease in the levels of T46 in the APP/PS1+PF treated group compared to the APP/PS1+vehicle group. Data are expressed as mean ± SD. Each experimental group consisted of n = 15 mice. §§§/***/### $p < 0.001$ vs. WT+Veh / APP/PS1+Veh / APP/PS1+PF.
